# Prognostic Value of Lymphocyte-Activation Gene 3 (LAG3) in Cancer: A Meta-Analysis

**DOI:** 10.3389/fonc.2019.01040

**Published:** 2019-10-15

**Authors:** Ramy R. Saleh, Paloma Peinado, Jesús Fuentes-Antrás, Pedro Pérez-Segura, Atanasio Pandiella, Eitan Amir, Alberto Ocaña

**Affiliations:** ^1^Division of Medical Oncology & Hematology, Department of Medicine, Princess Margaret Cancer Centre, University of Toronto, Toronto, ON, Canada; ^2^Experimental Therapeutics Unit, Medical Oncology Department, Hospital Clínico San Carlos, and IdISSC, Madrid, Spain; ^3^Centro de Investigación del Cáncer-CSIC, Salamanca, Spain; ^4^Centro de Investigación Biomédica en Red Cáncer (CIBERONC), Madrid, Spain; ^5^Centro Regional de Investigaciones Biomédicas, Castilla-La Mancha University (UCLM), Albacete, Spain

**Keywords:** LAG3, cancer, meta-analysis, lymphocyte activation gene 3, disease-free survival, overall survival

## Abstract

**Introduction:** Therapeutic targeting of inhibitors of the immune response has reached the clinical setting. Inhibitors of the novel receptor LAG3, which negatively regulates T-cell activation, are under investigation. Here we explore the presence and prognostic role of LAG3 in cancer.

**Methods:** A systematic search of electronic databases identified publications exploring the effect of LAG3 on overall survival (OS) and (for early-stage cancers) disease-free survival (DFS). Hazard ratios (HR) were pooled in a meta-analysis using generic inverse-variance and random effect modeling. Subgroup analyses were conducted based on disease site and tumor type.

**Results:** Fifteen studies met the inclusion criteria. LAG3 was associated with better overall survival [HR 0.81, 95% confidence interval (CI) 0.66–0.99; *P* = 0.04], with subgroup analysis showing no significant differences between disease-site subgroups. The beneficial effect of LAG3 on OS was of greater magnitude in early-stage malignancies (HR 0.73, 95% CI 0.60–0.88) than in the metastatic setting (HR 1.20, 95% CI 0.70–2.05), but this difference was not statistically significant (subgroup difference *p* = 0.18). LAG3 did not have a significant association with DFS [HR 1.02, 95% confidence interval (CI) 0.77–1.37; *P* = 0.87], with subgroup analysis showing worse DFS in patients with lymphoma and improved DFS in those with breast cancer.

**Conclusions:** High expression of LAG3 is associated with favorable overall survival in several solid tumors. A trend toward an association in early-stage disease suggests the importance of immune surveillance in this setting.

## Introduction

Inhibiting receptor downregulators of the immune response has become an attractive therapeutic approach, with successful results in some cancers (1). Inhibition of some of these receptors, including cytotoxic T-lymphocyte-associated protein-4 (CTLA-4) and programmed cell death-1 (PD-1) and its ligand PD-L1, has been shown to have anti-tumoral effects by inducing an effector response on CD8+ T cells (1–4). Compounds of this family have been approved in different indications, such as non-small cell lung (NSCLC), head and neck, bladder, and triple-negative breast cancers (5–9). In some but not all tumors, the presence of PD-L1-expression or markers associated with genomic instability such as tumor mutational burden or neo-antigen load predicts response to checkpoint inhibitors (5, 10–12).

For immune checkpoint inhibitors to be active, tumors must be in an inflamed or pre-activated state; so-called “hot tumors” (13). In this context, the presence of tumor-infiltrating lymphocytes (TILs) is necessary for adequate immune response (14, 15). Other co-inhibitory receptors have been described, including T-cell immunoglobulin and mucin-domain containing-3 (TIM-3) or the lymphocyte-activation gene-3 (LAG3) (16, 17). LAG3 is a transmembrane protein with structural homology to the CD4 co-receptor. It is found in activated CD4^+^ T cells, T-regulator cell, Tr1 cells, activated CD8^+^ T cells, natural killer cells, dendritic cells, B cells, and exhausted effector T cells (17, 18). The ligands for LAG3 include MHC class II molecules, galectin-3, liver sinusoidal endothelial cell lectin, and fibrinogen-like protein I (18, 19). Although the precise molecular mechanisms remain elusive, LAG3 negatively regulates the proliferation, activation, and effector function of T cells. To date, it is regarded as one of the multiple immune-checkpoint inhibitors that can promote immune tolerance through T-cell dysfunction and exhaustion (17, 18). Such negative effects seem to rely on the direct inhibition of CD3-mediated T-cell proliferation, cytokine production, and calcium flux (20). However, its suppressive effects are less apparent than those elicited by PD-1 or CTLA-4 in preclinical studies, suggesting the potential for a better safety profile (21, 22). Given its role as a negative regulator of T-cell activation, strategies aiming to inhibit its action are currently in clinical development (17, 23, 24). For both TIM-3 and LAG3, therapeutic antibodies are in clinical development, mainly in combination with inhibitors of PD-1 or PD-L1 (25).

The presence of TILs within the tumor is associated with a favorable outcome, demonstrating that the existence of an immune-activated state predicts an improved prognosis as well as response to immunotherapy (15, 26). In addition, the expression of PD-1 and PD-L1 is associated with a good outcome irrespective of treatment with immune checkpoint inhibitors, supporting the importance of the adaptive immune response (27–29). In this article, we aim to explore the frequency of expression and prognostic role of LAG3. We hypothesized that the expression of LAG3 would be associated with worse outcomes due to its inhibitory effects on the immune response.

## Methods

### Data Sources and Searches

This analysis was conducted following the Preferred Reporting Items for Systematic Reviews and Meta-analyses (PRISMA) guidelines. An electronic search of MEDLINE (host: Pubmed) from 1946 to March 30th, 2019 was performed using the search terms: “lymphocyte activation gene 3” or “LAG3” and “Cancer,” limiting results to studies in humans. Citation lists of the retrieved articles were screened manually to ensure the sensitivity of the search strategy.

### Study Selection

Eligibility criteria for studies included: (i) studies of humans (adults and children); (ii) patients with hematological or solid tumors; (iii) reporting of a hazard ratio (HR) for overall survival (OS) and/or disease-free survival (DFS; defined as the length of time from primary treatment of an early-stage cancer to death or any signs or symptoms of recurrent cancer) or survival curves allowing estimation of the HR for OS or DFS; (iv) availability as a full-text publication; (v) clinical trials, cohort, or case-control studies; and (vi) English language publication. Case reports, conference abstracts, and letters to editors were excluded. The titles identified by the initial search were evaluated, and potentially relevant publications were retrieved in full. Two authors (JFA and PP) reviewed the full articles independently for eligibility. Disagreements were resolved by consensus.

### Data Extraction

The following data were collected from the included studies using a predesigned abstraction form: name of first author, year of publication, journal, number of patients included in analysis, primary malignancy, protein expression, methods used for the evaluation of LAG3, and cut-off used for defining LAG3 intensity. Data extraction was performed by one author (RS). The outcome of interest was OS in patients both with and without LAG3-expression as defined by the individual studies. The HR for OS was extracted whenever available. In cases where the HR was not reported, it was estimated from survival curves for OS using the methods described by Parmar et al. (30). We applied a hierarchal approach to the collection of HRs, preferring those reported from multivariable analyses to univariable HR, and preferring both over HRs estimated from survival plots.

### Data Synthesis and Statistical Analysis

The extracted data were pooled using RevMan 5.3 analysis software (Cochrane Collaboration, Copenhagen, Denmark). Estimates for HRs were pooled and weighted by generic inverse variance and computed by random effect modeling. Statistical heterogeneity was assessed using the Cochran's Q and I^2^ statistics. Subgroup analyses were performed for different disease sites. Differences between the subgroups were assessed using methods described by Deeks et al. (31). Sensitivity analyses were performed, excluding studies in pediatric tumors and when LAG3-expression was evaluated using ELISA rather than immunohistochemistry (IHC) or next-generation sequencing (NGS). NGS sub-analysis was done between genetic variants A/G and G/G, with A/A as the referent group. A *post-hoc* exploratory analysis was performed to evaluate associations between the expression of LAG3 and other immune markers, including tumor mutational burden (TMB) and neoantigen burden. All of the statistical tests were two-sided, and statistical significance was defined as *p* < 0.05. No correction was applied for multiple statistical testing.

## Results

**Figure 1:**
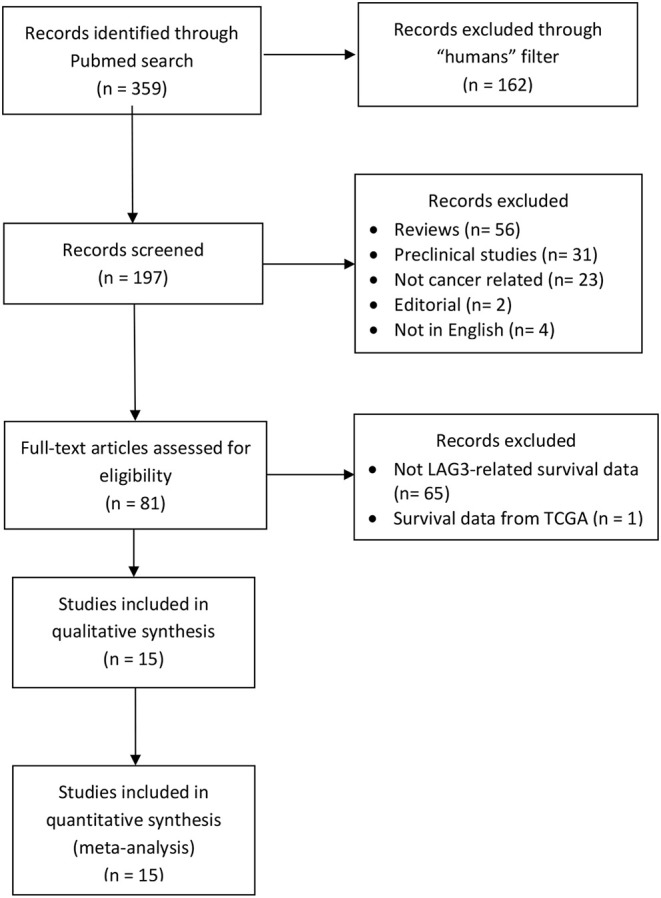
PRISMA of the study selection process for LAG3.

Fifteen retrospective studies comprising 6,306 patients were identified (Figure 1) (32–46). The characteristics of the included studies are listed in Table 1. LAG3 was reported as positive in 1,868 patients (31%). Fourteen studies explored the prognostic influence of LAG3 in adults, and one study was performed in a pediatric population. Two studies did not report OS outcomes, and five studies did not report DFS. The eligible studies explore outcomes in patients with breast, ovarian, gastric, lymphoma, NSCLC, colorectal, and renal cancers as well as pediatric neuroblastoma. Only two studies reported the use of neoadjuvant therapy, which was based on taxane/platinum combinations for breast cancer patients and 5-fluorouracil ± bevacizumab for colorectal cancer patients (32, 34). The eligible studies either did not deliver neoadjuvant therapy (*n* = 4) or excluded patients receiving pre-operative systemic therapy (*n* = 7) (36, 38, 40–42, 44, 46). Only two patients in the cohort of neuroblastoma were exposed to adjuvant, IL-2-based, immunotherapy (37). As for the rest of the studies, data on the treatment regimens was either not reported or details were incomplete, thereby not allowing additional investigation.

**Table 1:** Characteristics of included studies regarding LAG3.

**No**.	**Year**	**N**	**LAG3 +**	**Tumor**	**Pop**.	**Setting**	**Method**	**Agent**	**LAG-3-positive cut-off**	**Method HR reported**	**Outcomes included**
1	2017	2921	327 (11%)	Breast	Adult	Early	IHC (Ventana)	Clone 17B4 (Abcam, 1:100)	Cutoff: > 1/0.3 mm^2^	Multivariate	DFS OS
2	2018	41	39 (95%)	ENKTL nasal type	Adult	Early	IHC (SP)	ab180187 (Abcam, 1:100)	Moderate and strong intensities	Calculated	DFS
3	2017	139	36 (26%)	NSCLC	Adult	Early	IHC (Ventana)	EPR4392 (Abcam, 1:1000)	Cutoff > 20%	Calculated	DFS OS
4	2016	363	49 (14%)	TNBC	Adult	Early	IHC (Dako)	anti-LAG-3 (1:200, clone 17B4, LS Bio)	≥5%	Univariable	DFS OS
5	2017	553	325 (58%)	NSCLC	Adult	Early	IHC (Ventana)	D2G4O (Danvers, MA) 1:50	Mean core score: intraepithelial > 0, stromal > 0.5	Multivariate	DFS
6	2018	89	12 (14%)	CRC MSI-H	Adult	Early	IHC (Ventana)	Anti-LAG3 (1:100; Abcam)	Moderate-strong intensity in > 5% of cells	Multivariate	DFS
7	2018	308	N/A	Gastric	Adult	Early	ELISA	Wuhan USCN Sciences Co, 1:5	Cut-off point: 378.33 ng/mL	Calculated	OS
8	2015	80	9 (11%)	Renal	Adult	Mixed	IHC (Dako)	17B4	Positive cell density	Univariable	DFS, OS
9	2014	102	63 (62%)	CRC	Adult	Mixed	IHC	Ab (Abcam)	—	Calculated	OS
10	2006	246	116 (47%)	Breast HR+	Adult	Mixed	IHC	11E3 (IgG1) 17B4 (IgG1) mAb	> 120 pg/ml	Calculated	OS DFS
11	2017	439	277 (63%)	Gastric	Adult	Early	NGS	Genomic DNA extraction	LAG3 rs3782735	Multivariate	OS
12	2017	77	19 (24%)	Neuro blastoma	Peds	Mixed	IHC	EPR4392(2) Abcam	Mean positive cells in 10 fields/sample	Calculated	OS
13	2016	668	460 (69%)	CRC	Adult	Early	NGS	Genomic DNA QIAamp DNAeasy (Qiagen, Germany)	LAG3 rs3782735	Univariable	DFS, OS
14	2018	131	46 (35%)	Ovarian	Adult	Mixed	IHC	EPR4392 (Abcam, 1:100)	Immunoreactivity: low (<80%) or high (>80%)	Univariable	OS PFS
15	2015	149	90 (61%)	CRC	Adult	Metas.	NGS	Genomic DNA QIAamp DNAeasy (Qiagen, Germany)	LAG3 rs3782735	Multivariate	RFS, OS

*IHC, Immunohistochemistry; CLL, Chronic lymphocytic lymphoma; Ab, antibody; OS, Overall survival; RFS, Residual-free survival; NGS, Next-Generation Sequencing; Early, Early-stage disease (Stage I–III); Metas., Metastatic disease (Stage IV)*.

### Overall Survival

**Figure 2:**
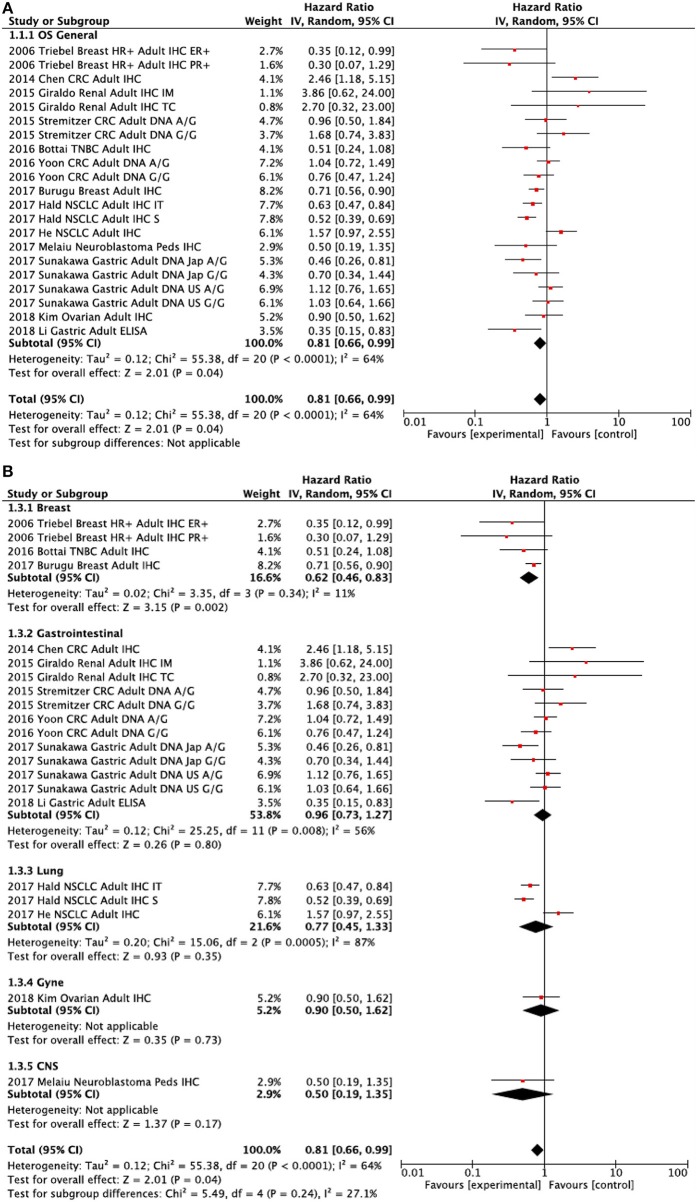
Forest plots showing hazard ratios for overall survival: LAG3 overall **(A)** and by subgroups based on disease site **(B)**. Hazard ratios for each study are represented by squares: the size of the square represents the weight of the study in the meta-analysis; the horizontal line passing through the square represents the 95% confidence interval. All statistical tests were two-sided. The diamonds represent the estimated pooled effect. Test for overall effect based on *z*-test. All *P*-values are two-sided. CI, confidence interval; OR, odds ratio. **(A)** LAG3 OS Overall. **(B)** LAG3 OS by disease site.

Data for the association between LAG3 and OS were reported in 13 studies. LAG3 was associated with better overall survival [HR 0.81, 95% confidence interval (CI) 0.66–0.99; *P* = 0.04, Figure 2A]. Heterogeneity was statistically significant (Cochran Q *P* < 0.001, I^2^ = 64%). Subgroup analysis showed that there were no significant differences between disease-site subgroups (Subgroup difference *P* = 0.24, Figure 2B). There was no significant difference between testing for LAG3 using IHC or DNA extraction (HR 0.79, 95% CI 0.58–1.07 vs. HR 0.91, 95% CI 0.73–1.14; subgroup difference *P* = 0.45). There was also no significant difference between genetic variants A/G and G/G relative to the A/A control group (subgroup difference *P* = 0.83). The beneficial effect of LAG3 on OS was of greater magnitude in early-stage malignancies (HR 0.73, 95% CI 0.60–0.88) than in the metastatic setting (HR 1.20, 95% CI 0.70–2.05), but this difference did not meet the statistical significance requirement (subgroup difference *p* = 0.18). Subgroup analysis showed that there was a greater magnitude of favorable prognosis with LAG3 expression in terms of OS when HRs were extracted rather than estimated (calculated HR 0.60, 95% CI 0.40–0.91, extracted HR 0.92, 95% CI 0.75–1.12). This difference approached but did not reach statistical significance (*p* for difference = 0.07).

### Disease-Free Survival

**Figure 3:**
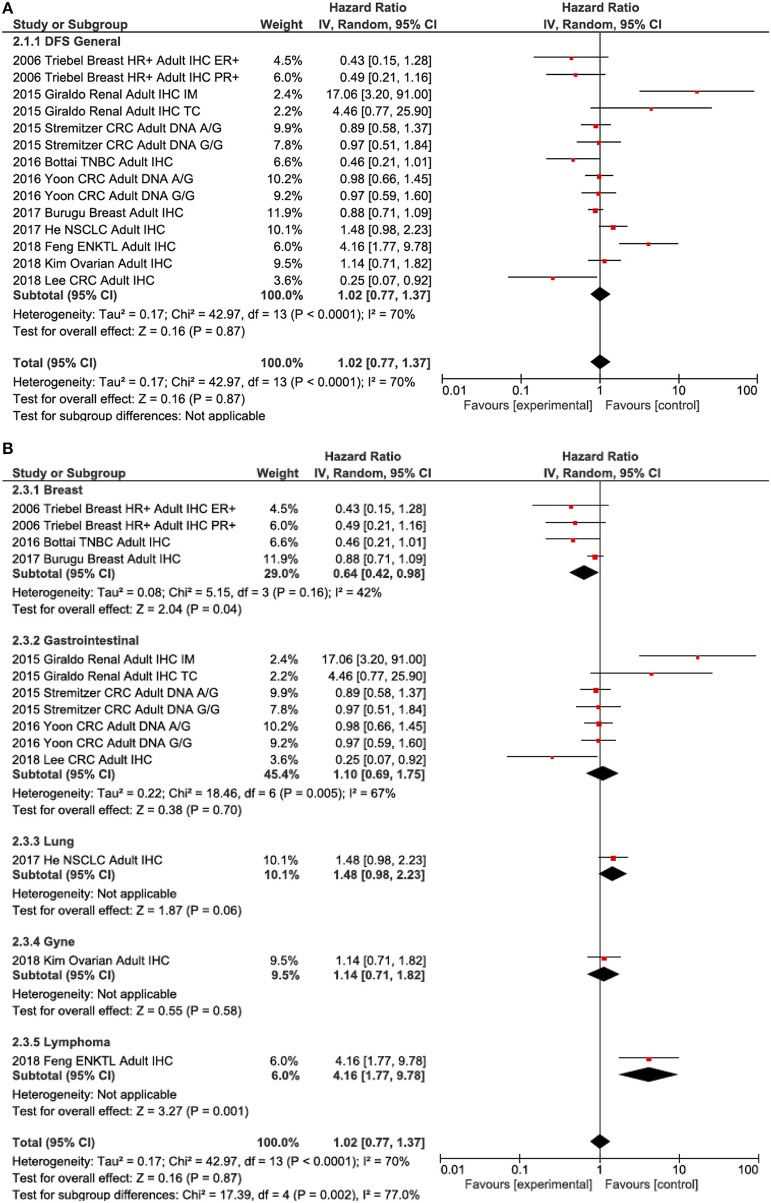
Forest plots showing hazard ratios for disease-free survival (DFS): LAG3 overall **(A)** and by subgroups based on disease site **(B)**. Hazard ratios for each study are represented by squares: the size of the square represents the weight of the study in the meta-analysis; the horizontal line passing through the square represents the 95% confidence interval. All statistical tests were two-sided. The diamonds represent the estimated pooled effect. Test for overall effect based on z-test. All *P*-values are two-sided. CI, confidence interval; OR, odds ratio. **(A)** LAG3 DFS overall. **(B)** LAG3 DFS by disease site.

Data for the association between LAG3 and disease-free survival (DFS) were reported in 10 out of 15 studies that included early-stage cancers. LAG3 did not exhibit a significant association with DFS [HR 1.02, 95% confidence interval (CI) 0.77–1.37; *P* = 0.87, Figure 3A]. Heterogeneity was statistically significant (Cochran Q *P* < 0.001, I^2^ = 70%). There was an association with worse DFS for lymphoma (HR 4.16, 95% CI 1.77–9.78, p = 0.001) and, to a lesser extent, NSCLC (HR 1.48, 95% CI 0.98–2.23, Figure 3B). Improved DFS was noted in the breast cancer group (HR 0.64, 95% CI 0.42–0.98). Excluding lymphoma and breast cancer, there was no significant difference between the remaining tumors (Subgroup difference *P* = 0.57), but heterogeneity remained statistically significant (Cochran Q *P* = 0.006, I^2^ = 63%). There was a modest and non-significant association between LAG3 and improved DFS in early-stage malignancies (HR 0.82, 95% CI 0.62–1.08) compared to the metastatic setting (HR 0.91, 95% CI 0.64–1.31), but this difference did not meet statistical significance requirements (subgroup difference *p* = 0.17). Again, subgroup analysis showed that there was a borderline significant difference in the prognostic value of LAG3 expression in terms of DFS based on whether HRs were extracted or estimated (calculated HR 1.12, 95% CI 0.46–2.72, extracted HR 0.96, 95% CI 0.72–1.27, *p* = 0.07).

### Correlation of LAG3 With Other Immunological Markers

The analysis of potential associations between LAG3 expression in TILs and other biomarkers in our work was hindered by the small number of studies providing combined data on PD-1, PD-L1, CD8, TIM3, and TMB. In a cohort of breast cancer patients, PD-L1, PD-1, and CD8 were positive in 53, 61, and 26% of LAG3^+^ TILs, respectively. In one study, concurrent infiltration of LAG3^+^ and CD8^+^ TILs in ER- breast cancer patients was associated with significantly longer DFS (HR 0.49, 95% 0.32–0.74) (32). Of note, LAG3^+^/PD-L1^+^ TILs have been identified in 15% of patients with triple-negative breast cancer (45). In an NSCLC cohort, PD-L1 and PD-1 were expressed in 47 and 70% of LAG3^+^ TILs, respectively. Up to 31% of NSCLC with LAG3^+^ TILs also expressed PD-L1 and, of note, the group of tumors without expression of either PD-L1- or LAG3- TILs showed longer relapse-free survival (2.09 years) than the group with PD-L1+ tumor cells and LAG3^+^ TILs (0.67 years) (46). Furthermore, significant positive correlations have been reported in renal cell carcinoma samples between LAG3 and CD8 and PD-1, indicating not only the coexistence of biomarkers but proportional variations (41). Finally, LAG3 and TIM3 appeared to be co-expressed in up to 71% of cases of extranodal nasal NK/T cell lymphoma (33). There were no data included on TMB or neoantigen burden.

## Discussion

In the present article, we describe the frequency of expression and prognostic value of LAG3 in several tumors. We find the presence of LAG3 to be associated with better OS. In the included studies, the effect was consistent in different tumor types. While, on average, no effect on DFS was observed, there was significant heterogeneity in effect between different disease sites. Of note as an interesting finding is the marked association in early-stage disease, which suggests an immunologic role in minimal residual disease.

LAG3 is a co-inhibitory receptor that represses the effector response of cytotoxic T cells (23, 24). In this context, several therapies are currently in clinical development to inhibit its activation, therefore facilitating an immune response (23). Synergy has been reported with the combination of LAG3 and PD-1 inhibition in murine models of melanoma, colorectal cancer, liver cancer, and fibrosarcoma, achieving responses in tumors largely resistant to single-agent immunotherapy (47). Also, CTLA-4 inhibition has been found to elicit an increase in the frequency of LAG3^+^ TILs in melanoma patients (48). These findings, together with a better safety profile, underscore the clinical interest of targeting LAG3 either alone or in combination with other immune checkpoint inhibitors. It has been demonstrated that concomitant treatment with LAG3 inhibitors with anti-PD-1 or PD-L1 can produce an enhanced effect, and, in this context, ongoing clinical studies are evaluating these combinations (2).

No difference was found between the methods used for the analysis of LAG3 expression or the different genetic variants defining expression. This suggests the sensitivity of the observed effect. Significant heterogeneity was observed among different tumors, and this may be a reflection of the heterogeneity observed in the immune response and in the presence of TILs in the different tumor types (49). This mirrors the discrepancy between tumors in the objective responses observed when checkpoint inhibitors are administered and the fact that response is not associated consistently with the expression of PD1 or PD-L1 in all tumor types (50).

An interesting finding is the association of LAG3 with a better outcome in the early-stage disease. It is well-established that the immune system plays a central role in avoiding long-term relapses in early-stage tumors (51). Although this effect is mediated by mechanisms that are not well-described, the presence of an active immune state contributes to the maintenance of cells in a quiescent state (51). In our study, we unexpectedly identified a better outcome with LAG3 in early-stage tumors. We are not aware of prior data reporting this association.

An association between the expression of immune inhibitory molecules such as PD-L1 and CTLA-4 and improved tumor outcomes has been described previously (29, 52, 53). Such an association seems paradoxical, since PD-L1, CTLA-4, and LAG3 elicit immunosuppressive responses and facilitate tumor escape. However, the upregulation of these molecules may initiate a negative feedback mechanism that creates an active immune environment in an inflamed tumor, which leads to an improved prognosis. Indeed, these markers usually overlap with CD8, which reflects an active host immunity and has established prognostic value (54). The expression of LAG3 was associated significantly with expression of PD-L1 and CD8 in the only two studies reporting such data (32, 41). More research is required to confirm these results.

The early stages of disease have been linked to a more intact host immunity and also to reduced tumor clonal heterogeneity. There is growing evidence that neoantigen heterogeneity may negatively influence immune surveillance and prognosis (55). Whether early-stage cancers have a more intact immune response due to a reduced pool of clonal neoantigens to target is a hypothesis that warrants investigation.

Contrary to our findings, the expression of other immune inhibitory receptors such as TIM3 has been associated with a worse prognosis (56), although no analysis based on early- or advanced-stage disease was performed. The results with LAG3 are more consistent with data observed with PD1 and PD-L1, which demonstrates a clear favorable prognosis when these markers are expressed (27–29).

The association between LAG3^+^ and other immunological biomarkers could not be addressed systematically in our work because of data scarcity. However, an association with biomarker aggregation is suggested by some studies in breast, NSCLC, and renal cancer patients (32, 41, 46). LAG3 and PD-1 have been found to be co-expressed consistently on both CD4^+^ and CD8^+^ TILs in several murine cancer models. Additionally, the co-inhibition of LAG3 and PD-1 elicits improved anti-tumor CD8^+^ T-cell responses (47). Additional data are needed to determine the impact on survival of aggregated biomarker expression in TILs and/or tumor cells. Similarly, an association has been described recently between tumors with LAG3^+^ TILs and a higher TMB based on data from the Cancer Genome Atlas (57). Unfortunately, none of the studies included in the current analysis reported data on TMB. While heterogenous and immature, this body of work suggests a functional interplay between immune markers and TMB. This warrants further research, especially regarding the potential for this interplay to provide predictive or prognostic value.

This study has limitations. This is a retrospective analysis of published articles. Therefore, it is susceptible to publication bias and also relies on summary data, not individual patient data. Furthermore, for some included studies (6 of 15), we estimated HR from survival plots, as it was not reported in the individual articles. Subgroup analysis showed that there was no significant difference in the prognostic value of LAG3 expression based on whether HRs were extracted or estimated. Finally, only one cohort reported data on hematologic malignancy and one on a pediatric population, so additional data on the prognostic value of LAG3 expression in hematological and pediatric malignancies is warranted.

In conclusion, we report the prognostic role of LAG3 in several tumors, suggesting that high expression is associated with a favorable outcome, particularly in terms of OS. The trend toward an association with outcome in early-stage disease supports the importance of immune surveillance in the setting of minimal residual disease.

## Author Contributions

AO: concept and design. JF-A, PP, and RS: collection and assembly of data. EA and RS: data analysis and interpretation. All authors: manuscript writing and final approval of manuscript.

### Conflict of Interest

EA reports personal fees from Genentech/Roche, personal fees from Apobiologix, personal fees from Myriad Genetics, personal fees from Agendia, outside the submitted work. AO reports personal fees from Entrechem, Servier, and Daiichi-Sankyo outside the submitted work. AP reports personal fees from Daiichi-Sankyo outside the submitted work. PP-S reports personal fees from Merck and MSD outside the submitted work. The remaining authors declare that the research was conducted in the absence of any commercial or financial relationships that could be construed as a potential conflict of interest.
